# Influence of Intraaortic Balloon Pump Counterpulsation on Transesophageal Echocardiography Derived Determinants of Diastolic Function

**DOI:** 10.1371/journal.pone.0118788

**Published:** 2015-03-04

**Authors:** Martina Nowak-Machen, Jan N. Hilberath, Peter Rosenberger, Eckhard Schmid, Stavros G. Memtsoudis, Johannes Angermair, Jayshree K. Tuli, Stanton K. Shernan

**Affiliations:** 1 Klinik für Anaesthesiologie und Intensivmedizin, Universitätsklinikum Tübingen, Germany; 2 Department of Anesthesiology, Perioperative and Pain Medicine, Brigham and Women’s Hospital, Harvard Medical School, Boston, Massachusetts, United States of America; 3 Department of Anesthesiology, Hospital for Special Surgery, Weill Cornell Medical College, New York, New York, United States of America; 4 Department of Statistics, University of Toronto, Toronto, Canada; University of Colorado Denver, UNITED STATES

## Abstract

**Introduction:**

Intraaortic balloon pump counterpulsation (IABP) is often used in patients with acute coronary syndrome for its favourable effects on left ventricular (LV) systolic function and coronary perfusion. However, the effects of IABP on LV diastolic function have not been comprehensively investigated. Acute diastolic dysfunction has been linked to increased morbidity and mortality. The aim of this study was to examine the influence of IABP on LV diastolic dysfunction using standard TEE derived parameters.

**Methods:**

Intraoperative TEE was performed in 10 patients (mean age 65 ± 11 yrs) undergoing urgent coronary artery bypass graft surgery (CABG), who had received an IABP preoperatively. TEE derived measures of diastolic dysfunction included early to late transmitral Doppler inflow velocity ratio (E/A), deceleration time (Dt), pulmonary venous systolic to diastolic Doppler velocity ratio (S/D), transmitral propagation velocity (Vp), and the ratio of early to late mitral annular tissue Doppler velocities (e’/a’). Statistical analyses included the Wilcoxon Sign-Rank test, and a p<0.05 was considered significant.

**Results:**

Transmitral inflow E/A ratios increased significantly from 0.86 to 1.07 (p < 0.05), while Dt decreased significantly from 218 to 180 ms (p < 0.05) with the use of IABP. Significant increases in Vp (34 cm/s to 43 cm/s; p < 0.05), and e’/a’ (0.58 to 0.71; p < 0.05) suggested a favourable influence of intraaortic counterpulsation on diastolic function.

**Conclusion:**

The use of perioperative IABP significantly improves TEE derived parameters of diastolic function consistent with a favourable impact on LV relaxation in cardiac surgery patients undergoing CABG.

## Introduction

Congestive heart failure (CHF) remains a major public health concern that is associated with markedly increased mortality. A significant proportion of patients with CHF demonstrate diastolic dysfunction with a normal systolic ejection fraction [1]. Diastolic dysfunction is defined as the inability of the ventricle to fill to a normal end-diastolic volume, both during exercise as well as at rest, without a significant increase in left atrial pressure [2]. Diastolic dysfunction has been reported in 44–75% of cardiac surgical patients prior to initiating cardio-pulmonary bypass (CPB) [3–5]. While diastolic heart failure annual mortality varies between 9–28%, which is up to four-fold that of disease-free subjects [6], it has also been linked to increased postoperative mortality or morbidity after cardiac surgery, increased difficulty in weaning from cardio-pulmonary bypass (CPB) and is a predictor of postoperative hemodynamic instability [2,3,7]. Following coronary artery bypass grafting (CABG), diastolic function frequently deteriorates at least temporarily [8], and may persist for at least the first several postoperative hours [2,9]. Thus, the diagnosis and management of perioperative diastolic dysfunction remains a topic of significant importance [1,10].

Intraaortic balloon pumping (IABP) was first described by Moulopoulos et al. in 1962 and has since become the most widely used temporary cardiac assist device, most frequently indicated for cardiogenic shock, hemodynamic support during catheterization and/or angioplasty, as well as for refractory post-myocardial infarction or unstable angina [11,12]. IABP has also been utilized prior to high-risk surgery as well as for difficulty weaning from CPB. The effects of IABP counterpulsation on systolic function have been well described and can be summarized as a favourable decrease in left ventricular (LV) end-systolic meridional wall stress mainly through afterload reduction during balloon deflation, and an increase in aortic diastolic pressure resulting in improved coronary perfusion during balloon inflation [13]. However, the effects of IABP counterpulsation on diastolic function have not been comprehensively investigated.

Transesophageal echocardiography (TEE) has become a widely available, practical and safe tool to evaluate patients with diastolic dysfunction [1,14]. Many TEE derived parameters such as transmitral inflow Doppler flow velocity profile assessment, E-wave deceleration time, tissue Doppler imaging of the mitral valve (MV) annulus and transmitral propagation velocity (Vp), have been proposed to assess LV compliance and the severity of diastolic dysfunction [1,15,16]. As the influence of IABP on perioperative diastolic dysfunction has not been thoroughly investigated, the goal of this study was to examine whether IABP counterpulsation influences TEE derived parameters of diastolic dysfunction in a population of patients undergoing CABG surgery.

## Patients and Methods

### Study Population

The data were collected as part of a prospective Institutional Review Board protocol at Brigham and Women’s Hospital, Harvard Medical School, Boston MA.

approved protocol with a waiver of informed consent. The study population consisted of hemodynamically stable patients scheduled for CABG surgery who had full support from an IABP (1:1), placed preoperatively for signs and symptoms of myocardial ischemia refractory to medical therapy. Exclusion criteria for this study included intraoperative TEE evidence of significant valvular dysfunction, or either pseudonormal or restrictive patterns of diastolic dysfunction as previously defined [17]. Patients who were not in sinus rhythm were also excluded from the study.

### Anesthesia, loading conditions and inotropic support

General anesthesia was conduced in all patients following the institutional standards using Fentanyl (3–5 mcg/kg), Etomidate (0.2–0,3 mg/kg) and Vecuronium (0,1 mg/kg) for induction and Fentanyl (up to 5 mcg/kg divided into multiple boli for the duration of the surgery) as well as Isoflurane (up to 1 MAC, guided by BIS monitoring). Patients did not receive more than 1,5l of i.v. crystalloid fluid total during surgery in addition to the CPB priming solution. On average patients received 500 ml of fluid before CPB and 500–1000 ml after CPB until the end of surgery. None of the enrolled patients needed inotropic support during the surgery (data not shown).

### Intraoperative Transesophageal Examination

Routine, comprehensive intraoperative TEE examinations were performed after induction of general anesthesia by National Board of Echocardiography diplomats. Immediately prior to aortic cannulation, the TEE acquired measures of diastolic function outlined below, were obtained as part of a standard comprehensive intraoperative TEE examination in patients with IABP support. During aortic cannulation, IABP support was paused per routine clinical protocol. Immediately before re-initiating IABP support, the acquisition of TEE measures of diastolic function were repeated again as part of a standard protocol in this patient population. All echocardiographic measurements were obtained over 3 heart beats, and the mean of these measurement used for analysis.

### Echocardiographic Measurements of Diastolic Dysfunction

Echocardiographic determinants of diastolic dysfunction were obtained according to ASE guidelines [4]. Specifically, transmitral inflow measurements (E/A) were obtained from pulse wave Doppler flow velocity profiles after placing the sample volume at the tip of the MV leaflets. The deceleration time (Dt) was determined by measuring the time from the peak of the mitral inflow E wave to the baseline. Pulmonary vein flow profile (S/D-ratio) was measured from pulsed-wave Doppler flow velocity profiles of the left upper pulmonary vein. Mitral annular tissue-Doppler measurements (e’/a’) were performed by placing the pulsed wave Doppler sample probe at the lateral corner of the mitral annulus. For determination of the propagation velocity (Vp), color M-mode was used in the midesophageal 4-chamber view at 0° rotation.

### Statistical Analysis

The data were processed using JMP Software (Cary, NC) for Statistical Analysis and figures were generated using Kaleidagraph (Reading, PA). Wilcoxon Sign ranked test was used and a p-value < 0.05 was considered significant.

## Results

### Patient Population

The study population consisted of 5 female and 5 male patients, median age of 64.7 years ± 11 years. All patients were hemodynamically stable, in sinus rhythm with a heart rate between 80–100 beats/minute at the time of TEE examination, and were not requiring pharmacological hemodynamic support.

### Transesophageal echocardiographic determinants of diastolic function

**Fig 1:**
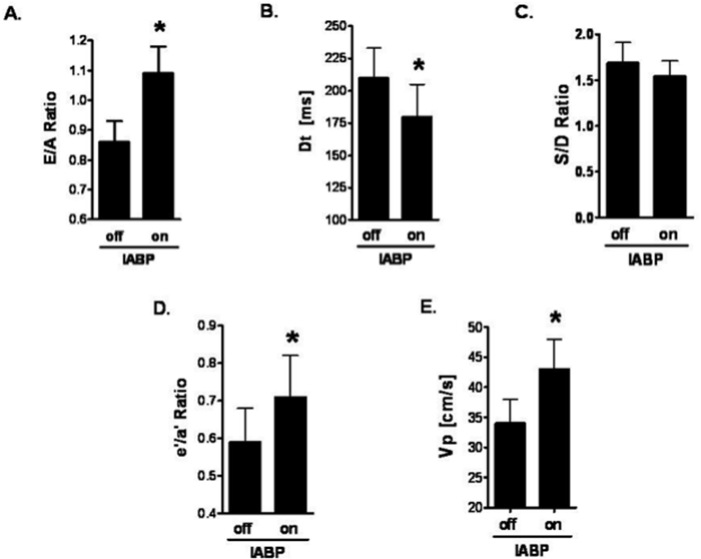
Intraoperative transesophageal echocardiographic measures of diastolic function without and with intraaortic balloon counterpulsation (IABP). A) Mitral valve Doppler flow velocity inflow ratio (E/A). B) Deceleration time (Dt) in milliseconds (ms). C) Pulmonary vein Doppler flow velocity systolic (S) to diastolic (D) ratio. D) Mitral annular tissue Doppler velocity ratio (e’/a’). E. Propagation velocity (Vp) in milliseconds (m/s). Values are Mean ± SD. P value < 0.05 was considered significant (*).

Parameters of transmitral inflow (E/A-ratio) increased significantly from 0.86 without IABP to 1.07 with IABP support (p < 0.05) (Fig. 1A). The deceleration time (Dt) decreased from 218 ms without IABP to 180 ms with IABP support (p < 0.05) (Fig. 1B). The S/D ratio (1.66 without IABP to 1.5 with IABP, p = 0.7) showed no significant difference between time points (Fig. 1C). The mitral annular tissue-Doppler ratio (e’/a’) increased significantly from 0.58 without IABP to 0.71 with IABP (p < 0.05) (Fig. 1D). In addition, the propagation velocity (Vp) increased significantly from 34 cm/s without IABP to 43 cm/s with IABP (p < 0.05) (Fig. 1E).

## Discussion

Intraaortic balloon counterpulsation (IABP) is the most commonly used form of temporary cardiac assist device, and is often used in cardiac surgical patients with acute coronary syndrome or to facilitate difficulty weaning from CPB. A significant number of critically ill patients experience clinically significant diastolic dysfunction, which increases their overall morbidity and mortality during and after cardiac surgery [2]. In this present study, we demonstrate that TEE derived determinants of LV diastolic function improved significantly with the use of IABP during CABG surgery. Thus, in addition to its known benefits on LV systolic function, IABP has the potential to improve LV diastolic dysfunction in cardiac surgery patients.

IABP related improvements in diastolic function may be related to both physiologic and mechanical factors. Kass et al. previously demonstrated that increased coronary perfusion during IABP support improves tissue elasticity, a major determinant of diastolic dysfunction [18,19]. Similarly, Gill et al. showed that the development of diastolic dysfunction was related to decreased coronary perfusion induced by recurrent coronary embolization [20]. Furthermore, metabolic disturbances and increased central blood volume both seem to be contributing factors for the development and continued prevalence of diastolic dysfunction [21]. Changes in preload and afterload are also known to significantly impact cardiac relaxation [20]. The impact of LV preload on ventricular relaxation was previously investigated by Komamura et al. who showed that increased LV preload impairs LV isovolumetric relaxation, and that baseline isovolumetric relaxation can be restored after normalizing loading conditions [22]. In addition to this important finding, Cheung at al. demonstrated a decrease in LV end-diastolic cross-sectional area during IABP support, suggesting that short-term IABP support decreases LV preload [13]. Furthermore, early experimental studies in LV failure showed that IABP acutely decreases left ventricular end-diastolic pressure [23], which is consistent with a decreased LV preload as previously suggested, or it could point towards improved diastolic filling mechanics and improved LV elasticity during IABP support. Thus, LV unloading, in addition to increased coronary perfusion pressure, could be one of the mechanisms leading to improved diastolic function under IABP support in our patient population.

The practical utility and value of echocardiography for the evaluation of diastolic function has been well established over the last two decades and has become an important focus for the development of practice guidelines [15]. The use of intraoperative TEE to diagnose perioperative diastolic dysfunction and its clinical relevance remains a topic of increasing importance [1]. However, hemodynamic volatility in the perioperative period may provide a challenging environment for the use of load-dependent echocardiographic measures of diastolic function including the transmitral E/A-ratio and pulmonary vein (S/D) Doppler flow velocity profiles [24]. Consequently, less load-dependent measures including tissue-Doppler ratio (e’/a’) and propagation velocity (Vp) may have greater value in the perioperative period [2,25–27]. Thus, we chose to include both conventional, known load-dependent and less-load dependent echocardiographic measures of diastolic function in our investigation.

While the impact of IABP on diastolic function has been reported, this concept has not been comprehensively investigated. Khir et al. studied 20 cardiac ICU patients within 36 hours post cardiac surgery, and evaluated left anterior descending (LAD) coronary artery and transmitral E-wave flow velocities, as well as LV long axis free-wall movement. Increased diastolic LAD coronary artery flow velocities and LV free-wall early diastolic lengthening velocity were consistent with improved diastolic function under IABP support when compared to control conditions without IABP [28]. While the results of this study are consistent with our data, the use of echocardiography was limited. Shimamoto et al. also investigated the effects of IABP on intraoperative TEE acquired mitral flow dynamics in 15 patients after CABG [29]. Similar to our results, IABP significantly decreased the ratio of the transmitral contraction phase (A wave) to rapid filling phase Doppler flow velocity integral (i.e., increased the E/A ratio). However, the less load-dependent echocardiographic measures of diastolic function including e’/a’ and Vp were not evaluated.

In our present study, the robust association between the use of IABP with an increase in the E/A ratio and a decrease in Dt, as well as increases in the less load-dependent e’/a’ and Vp towards more normal values, is consistent with an improvement in LV relaxation and a favourable impact of IABP on LV diastolic function perhaps by facilitating ventricular unloading. While a decrease in the S/D ratio would also have been consistent with a favourable effect, the absence of a statistically significant result may reflect the low sensitivity of this load-dependent parameter in the volatile hemodynamic environment of the operating room setting. Nonetheless, identifying high-risk patients preoperatively and monitoring their diastolic function intraoperatively may warrant the use of intra-aortic counterpulsation to facilitate separation from CPB.

Certain limitations of this presented work are worth noting including its small sample size and the absence of a control population to determine the potential clinical benefits of IABP associated improvements in perioperative diastolic function. Nonetheless, the demonstrated influence of IABP counterpulsation on the evaluated echocardiographic measurements of diastolic dysfunction appears favourable, and suggests that a larger study is warranted to elucidate a role of IABP in acute perioperative diastolic dysfunction.

In conclusion, IABP counterpulsation has a positive impact on TEE derived parameters of diastolic dysfunction in patients undergoing urgent CABG, in addition to the previously known beneficial effects on systolic dysfunction. The mechanisms seem to include a favourable direct effect on improving LV relaxation. Further investigation is warranted to determine whether IABP could become a complimentary part of multi-targeted approach towards therapeutic intervention in patients experiencing acute perioperative diastolic dysfunction.
